# Prevalance of *BRCA1* and *BRCA2* mutations in familial breast cancer patients in Lebanon

**DOI:** 10.1186/1897-4287-10-7

**Published:** 2012-06-19

**Authors:** Nadine Jalkh, Jinane Nassar-Slaba, Eliane Chouery, Nabiha Salem, Nancy Uhrchammer, Lisa Golmard, Domique Stoppa-Lyonnet, Yves-Jean Bignon, André Mégarbané

**Affiliations:** 1 Unité de Génétique Médicale et laboratoire associé INSERM à l’Unité UMR_S910, Université Saint-Joseph, Beirut, Lebanon; 2 Département d'Oncogénétique, Centre Jean Perrin, Clermont-Ferrand, France; 3 Génétique oncologique, Institut Curie-Hôpital, Inserm U830, Université Paris-Descartes, Paris, France; 4 Unité de Génétique Médicale. Faculté de Médecine, Université Saint-Joseph, 42, rue de Grenelle, Paris, 75007, France

**Keywords:** BRCA1, BRCA2, Breast cancer, Familial, Gene, Lebanon, Mutation

## Abstract

Breast cancer is the most prevalent malignancy in women in Western countries, currently accounting for one third of all female cancers. Familial aggregation is thought to account for 5–10 % of all BC cases, and germline mutations in *BRCA1* and *BRCA2* account for less of the half of these inherited cases. In Lebanon, breast cancer represents the principal death-causing malignancy among women, with 50 % of the cases diagnosed before the age of 50 years.

In order to study *BRCA1/2* mutation spectra in the Lebanese population, 72 unrelated patients with a reported family history of breast and/or ovarian cancers or with an early onset breast cancer were tested. Fluorescent direct sequencing of the entire coding region and intronic sequences flanking each exon was performed.

A total of 38 *BRCA1* and 40 *BRCA2* sequence variants were found. Seventeen of them were novel. Seven confirmed deleterious mutations were identified in 9 subjects providing a frequency of mutations of 12.5 %. Fifteen variants were considered of unknown clinical significance according to BIC and UMD-BRCA1/BRCA2 databases.

In conclusion, this study represents the first evaluation of the deleterious and unclassified genetic variants in the *BRCA1/2* genes found in a Lebanese population with a relatively high risk of breast cancer.

## Introduction

In the western countries the lifetime risk of developing breast cancer is around 12 %. About 5 %–10 % of breast cancer (BC) and ovarian cancer (OC) are hereditary and 30 %–50 % of these are due to mutations with an autosomal dominant inheritance [1]. Deleterious mutations in the *BRCA1* and *BRCA2* genes are the principal known cause of hereditary BC. They are localized on chromosomes 17q21 and 13q12 respectively [2,3]. They are found in 1/400-1/800 people in the general population [4-6].

For women with a personal or family history of breast and/or ovarian cancer, *BRCA1/2* full-sequencing and analysis for large genomic rearrangements (LGR) are routinely used to quantify the genetic component of cancer risk. The spectrum of mutations found in *BRCA1*/*2* depends on the studied population, ranging from few founder mutations in some, to a wide spectrum of mutations in others [7].

In Lebanon, breast cancer remains the most frequent type of cancer among women since 1960. In 2005, its age adjusted incidence rate was estimated at 76 new cases per 100.000 by the Public Health Ministry of Lebanon and it is still increasing (http://www.public-health.gov.lb). In France, during the same period, it was of 101 new cases per 100.000 according to the National Institute of Cancer (http://www.e-cancer.fr/). On the other hand, the median age at diagnosis for BC in Lebanon is 52.5 years, approximately 9 years younger than the European/North American median [8].

Given that a woman with breast cancer is more likely to carry a susceptibility gene mutation the younger she is at the time of diagnosis [5,9,10], one possibility is that the allelic frequency of high penetrance genes in the Lebanese population may be higher than that in Caucasian populations.

In order to determine the spectra and frequency of *BRCA1/2* mutations within the Lebanese population, we studied a cohort of 72 Lebanese unrelated patients with breast cancer.

## Subjects and Methods

### Participants

From 2001 to 2011, 72 unrelated patients were selected to undergo sequence DNA testing for mutations in *BRCA1/2*. They were referred from a wide variety of settings from all over the country, ranging from private physicians’ offices to major academic medical centers, because of elevated risk of a *BRCA1/2* mutation: a personal history of invasive breast cancer and at least one of the following criteria: A) breast cancer at any age at onset in ≥ 2 first- and/or second-degree relatives, B) breast cancer <50 years in a first- or second-degree relative, C) ovarian cancer in ≥ 2 first- and/or second-degree relatives, D) breast and ovarian cancer in ≥ 2 first- and/or second-degree relatives, E) both breast and ovarian cancer in a single first- or second-degree relative, F) male breast cancer.

Two cases in men with a personal history of sporadic breast cancer were also included.

#### *BRCA1 and BRCA2 analysis*

After an informed consent was signed, DNA was extracted from 10 ml of peripheral blood using standard methods [11]. All exons and > 50 bp of each flanking intron were amplified in a final volume of 50 μl containing 100 ng DNA, 0.25 mM dNTPs, 100 ng of each primer and 0.02 unit of Taq polymerase (Invitrogen Life Technologies, Carlsbad, CA, USA). PCR was performed in an ABI9700 thermocycler (Applied Biosystems, Foster City, CA) with initial denaturation at 94 °C for 5 min, followed by 35 cycles of (95 °C for 30s, specific annealing temperature for 30s, 72 °C for 30s), except for *BRCA1* exons 14 and 15 where 5 cycles were added to the initial 35 cycles. Primers sequences are available on request as well as annealing temperatures of each exon.

PCR products were purified using the illustra^TM^ GFX PCR DNA and Gel Band Purification Kit (GE Healthcare, Buckinghamshire, UK), and resuspended in 40 μl of deionized water. Both strands of the resultant products were sequenced using the BigDye® Terminator v1.1 Cycle Sequencing Kit (Applied Biosystems, Foster City, CA) under standard conditions. The labeled products were subjected to electrophoresis on an ABI3130 Genetic Analyzer sequencing system (Applied Biosystems, Foster City, CA, USA). Electropherograms were analyzed using Sequence Analysis Software version 5.2 (Applied Biosystems, Foster City, CA, USA) and compared to reference sequences (*BRCA1*: ADNg L78833; ADNc NM_007294.3 and *BRCA2*: ADNg Z74739; ADNc NM_000059.3) using ChromasPro version 1.5 (Technelysium, Queensland, Australia). All deleterious mutations were confirmed first on an independent second amplification and second on a second blood specimen.

Nucleotide numbering reflects cDNA numbering with +1 corresponding to the A of the ATG translation initiation codon in the reference sequences.

The effect of missense mutations was predicted using the BIC database (http://research.nhgri.nih.gov) and the UMD-BRCA1/BRCA2 databases and the prediction programs: SIFT (Sorting Intolerant From Tolerant) (http://sift.jcvi.org/www/SIFT_Blink_submit.html), PolyPhen (http://genetics.bwh.harvard.edu/pph/index.html), and Align-GVGD from the International Agency for research on Cancer (http://agvgd.iarc.fr) [12,13]. Prediction of splicing effect was performed with MaxEntScan using a 15 % variation threshold as proposed by Houdayer *et al.*[14,15].

Identified variations were classified into three categories: deleterious, unclassified variant, and neutral.

## Results

The mean age at diagnosis of BC for the seventy-two patients was 41 years old. Thirty-seven patients provided us with their histopathological results. Thirty-one patients had a positive estrogen-receptor (ER) and a positive progesterone-receptor (PR) disease, four patients had a positive ER and a negative PR disease and one patient had a negative ER and a positive PR disease. Only one patient had a triple negative disease and was not found to have any deleterious mutation.

**Figure 1:**
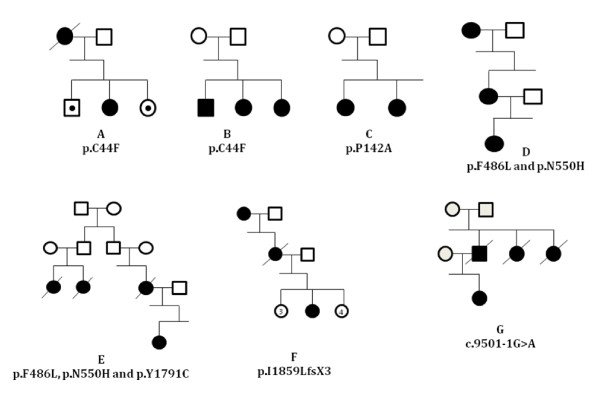
**Pedigrees of the families presenting*BRCA1*(A-E) and*BRCA2*(F,G) mutations. **Squares and circles with solid dark color indicate the affected individuals. The dots in the squares and circles with solid dark color indicate the affected individuals.

Within this cohort, we identified a total of 38 *BRCA1* and 40 *BRCA2* sequence variants, of which 17 (21.8 %) were novel (not reported in the databases BIC and UMD-BRCA1/BRCA2): 11 (14.1 %) in *BRCA1* and 6 (7.7 %) in *BRCA2* (Tables 1 and 2). Five disease-associated *BRCA1* mutations and two disease-associated *BRCA2* mutations were found in this cohort (Figure 1).

**Table 1:** **Sequence variants identified in the*BRCA1*gene**

**Nucleotide Change**	**AA change**	**Number of patients**	**Clinical Relevance (BIC)**	**Variation class^§^**	**Polyphen**	**SIFT**	**Align GVGD**
Missense variations							
250 G > T	C44F*	2	Unknown	5	+++	Not tolerated	Class 65
543 C > G	P142A*	1	Not reported	Not reported	++	Not tolerated	Class 0
585 C > A	L156I	1	Not reported	3	+	Not tolerated	Class 0
655A > G	Y179C†	2	Unknown	1	+++	Not tolerated	Class 45
1186A > G	Q356R	19	Unknown	1	+++	Not tolerated	Class 0
1575 T > C	F486L†	2	Unknown	1	+	Tolerated	Class 0
1767A > C	N550H†	2	Unknown	1	++	Not tolerated	Class 0
2196 G > A	D693N	11	No effect	1	+	Not tolerated	Class 0
2577A > G	K820E	1	Unknown	1	+	Tolerated	Class 0
2731 C > T	P871L	47	No effect	1	+	Tolerated	Class 0
3232A > G	E1038G	44	No effect	1	+	Not tolerated	Class 0
3238 G > A	S1040N	2	Unknown	1	+	Tolerated	Class 0
3646 T > A	V1176I	1	Unknown	Not reported	+	Tolerated	Class 0
3667A > G	K1183R	43	No effect	1	+	Tolerated	Class 0
4755 G > A	D1546N	1	Unknown	1	+	Tolerated	Class 0
4773 T > C	Y1552H	1	Not reported	3	++	Tolerated	Class 0
4956A > G	S1613G	45	No effect	1	+	Not tolerated	Class 0
5075 G > A	M1652I	2	Unknown	1	+	Tolerated	Class 0
Nonsense mutations							
5563 G > A	W1815X	2	Deleterious	5			
Synonyms							
1964 T > C	S615S	2	Not reported	Not reported			
2201 C > T	S694S	47	No effect	1			
2430 T > C	L771L	45	No effect	1			
4427 T > C	S1436S	44	Unknown	1			
5306 G > C	L1729L	1	Not reported	Not reported			
UTRs and Intronic variations							
66-67delTA	5’-UTR	29	Not reported	Not reported			
IVS2-91A > G		1	Not reported	Not reported			
IVS5 + 45 T > A		1	Not reported	Not reported			
IVS6 + 55 G > A		2	Not reported	3			
IVS7 + 15delTTC		6	Not reported	Not reported			
IVS7-34 C > T		17	No effect	1			
IVS8-58delT		10	No effect	1			
IVS11 + 106 G > A		64	Not reported	Not reported			
IVS13 + 117 G > A		8	Unknown	Not reported			
IVS14-63 C > G		25	Not reported	Not reported			
IVS18 + 66 G > A		44	No effect	1			
IVS19-26 G > A		2	Not reported	Not reported			
IVS22 + 68 T > C		1	Unknown	3			
c.5711 + 36 C > G	3’-UTR	1	Not reported	Not reported			

† Missense considered deleterious when associated.

*Missense considered deleterious.

Polyphen: +++ Variant predicted to be probably damaging, ++ Variant predicted to be possibly damaging, + Variant predicted to be benign.

UMD-BRCA1/2: 1 Variant considered neutral, 2 Variant considered likely-neutral, 3 Variant considered unclassified, 4 Variant considered likely-causal, 5 Variant considered causal.

^§^ in the UMD-BRCA1 database (http://www.umd.be/BRCA1/) (12) using classification system in five classes (13).

**Table 2:** **Sequence variants identified in the*BRCA2*gene**

**Nucleotide Change**	**AA change**	**Number of patients**	**Clinical Relevance (BIC)**	**Variation class^§^**	**Polyphen**	**SIFT**	**Align GVGD**
Frameshift mutations							
5804_5808delTTAA	I1859KfsX3	1	Deleterious	5			
c.9485-1 G > A*		1	Not reported	Not reported			
Missense variations							
353A > G	Y42C	1	Unknown	1	+++	Tolerated	Class 0
1093A > C	N289H	6	No effect	1	+	Not tolerated	Class 0
1342A > C	N372H	20	No effect	1	+	Tolerated	Class 0
3199A > G	N991D	2	Unknown	1	++	Tolerated	Class 0
5972 C > T	T1915M	2	Unknown	1	++	Tolerated	Class 0
6359 G > C	G2044A	1	Unknown	3	+	Tolerated	Class 0
6550 C > T	R2108C	1	Unknown	3	+++	Tolerated	Class 0
7625 C > T	A2466V	26	Unknown	1	+	Not tolerated	Class 0
9079 G > A	A2951T	1	No effect	2	+	Not tolerated	Class 55
Nonsense mutations							
10204A > T	K3326X	1	No effect	1			
Synonyms							
1593A > G	S455S	6	No effect	1			
2457 T > C	H743H	6	Unknown	1			
3624A > G	K1132K	16	No effect	1			
4035 T > C	V1269V	13	No effect	1			
4791A > G	L1521L	38	No effect	2			
5718 C > T	S1830S	1	Not reported	Not reported			
6741 C > G	V2171V	38	Unknown	1			
7470A > G	S2414S	13	No effect	1			
10338 G > A	R3370R	1	Unknown	1			
UTRs and Intronic variations							
203 G > A	5’UTR	16	No effect	Not reported			
IVS4 + 67A > C		6	Unknown	1			
IVS4 + 147 G > T		4	Not reported	Not reported			
IVS6 + 14 C > T		1	Unknown	3			
IVS7 + 183 T > A		21	Not reported	1			
IVS7-9A > G		1	Unknown	Not reported			
IVS8 + 56 C > T		8	Unknown	1			
IVS10-34 C > A		1	Not reported	3			
IVS10-51 G > T		1	Not reported	1			
IVS11 + 80delTTAA		10	Unknown	3			
IVS12-120 T > C		37	Not reported	3			
IVS13 + 133insATTATAAAA		5	Not reported	Not reported			
IVS13 + 273 G > A		2	Not reported	Not reported			
IVS14 + 53 C > T		4	No effect	Not reported			
IVS16-14 T > C		25	No effect	1			
IVS21-66 T > C		25	No effect	1			
IVS22 + 53 C > G		1	Not reported	Not reported			
IVS24-16 T > C		1	Unknown	1			
10485 + 105A > C	3’UTR	15	Not reported	Not reported			

*Missense considered deleterious.

Polyphen: +++ Variant predicted to be probably damaging, ++ Variant predicted to be possibly damaging, + Variant predicted to be benign.

UMD-BRCA1/2: 1 Variant considered neutral, 2 Variant considered likely-neutral, 3 Variant considered unclassified, 4 Variant considered likely-causal, 5 Variant considered causal.

^§^ in the UMD-BRCA1 database (http://www.umd.be/BRCA1/) (12) using classification system in five classes (13).

Six out of the 38 variants found in *BRCA1* (15.8 %) were found to be of uncertain clinical significance according to BIC and UMD. Nineteen variants were known polymorphisms, 14 were intronic variants, 1 was a nonsense mutation (p.W1815X), and 23 missense variations (Table 1), out of which two were found of interest: p.C44F and p.P142A [16,17]. The first one was associated with a strong family history of breast and ovarian cancer in two unrelated families and the second one was found in a large family with many affected women of different age of onset. One patient presented the association of the three missense variations: p.Y179C, p.F486L and p.N550H, and one patient the variations p.N550H and p.F486L. In 41 patients (57 %) a haplotype presenting the combination of the following variations: p.S694S, p.L771L, p.P871L, p.E1038G, p.K1183R, p.S1436S, p.S1613G and IVS18 + 66 G > A, was found. In 9 of those, an additional variation, p.Q356R, was found. The *BRCA1* p.Y1552H variation was the only mutation associated to the frequent haplotype found in our population.

Nine out of the 40 variants found in *BRCA2* (22.5 %) were of uncertain clinical significance according to BIC and UMD databases. Twenty-three were known polymorphisms out of which one nonsense variation (p.K3326X). Eighteen variants were missense variations, 19 intronic variants, and 2 frameshift mutations (Table 2). For the latter, the first one, c.5576_5579delTTAA in exon 11, is a deletion of 4 bases pairs leading to a frameshift and a stop codon three amino acids further down (p.I1859KfsX3). This mutation was identified in a patient diagnosed at the age of 51 years and was considered as a truncating mutation. The family history of this individual included a breast cancer diagnosed in the mother at 63 years. The second mutation is at position c.9485-1 G > A leading to exon 26 skipping. It was identified in a patient diagnosed at the age of 33 years. The family history of this individual included a breast cancer diagnosis of the father and the paternal aunt.

No variant was predicted to affect splicing except the *BRCA2* c.9485-1 G > A mutation which disrupts an acceptor splice site consensus sequence.

## Discussion

In the present study, we have identified *BRCA1* and *BRCA2* germline deleterious mutations in 9 carriers among a cohort of 72 unrelated Lebanese patients with BC, providing a frequency of mutations which represents a prevalence of 12.5 %.

The low prevalence of mutations found in our population in comparison to other ones [5,18-20], can be explained either by the genetic testing criteria, or by the possibility that some mutations were missed since we could not look for deletions or duplications of entire exons. Consanguinity might be also an equivocal risk-modifying factor [21,22]. Indeed, the Lebanese population has a relatively high rate of consanguinity (around 15–20 %), and association studies on consanguinity and breast cancer, and the frequency of *BRCA*1 and *BRCA2* in highly consanguineous populations pointed that consanguinity might lead to decreased incidence of breast cancer [23]. Nevertheless, other studies obtained contradictory results and the question remains open [23].

Our findings could not contribute in understanding the cause of the high incidence of BC in Lebanon and the lower age of onset observed compared to western countries. The low age of onset was also reported in other Asian countries, and Arab countries in the Middle East [24-26]. It may be explained by the differences in exposure to female hormones, diet, physical activity, or other factors. For instance, the “Westernization” of these populations is due to delayed childbearing, lower parity, reduced breast-feeding, decreased exercise and dietary changes [24]. It can lead to a ‘cohort effect’ where the younger cohort have increased breast cancer risk compared to their mothers and grandmothers, thus giving rise to an apparent lower mean age of onset [25].

In *BRCA1*, a haplotype presenting the combination of the variations: p.S694S, p.L771L, p.P871L, p.E1038G, p.K1183R, p.S1436S, p.S1613G and IVS18 + 66 G > A was very often found in the Lebanese population. In fact, Dunning *et al*. reported a close to complete allelic association between the alleles at the 871, 1038 and 1613 residues [27]. Freedman *et al.* also reported this haplotype, except for the p.S694S variation, with a frequency of 32 % [28]. The latter study found no significant differences of frequencies between patients and control groups [28].

A major problem, the pathogenic status of several sequence variants that are novel or considered of unknown clinical significance, remains unresolved. For instance, a patient had the association of three missense variations: p.Y179C, p.F486L and p.N550H. Taken separately these variations were considered few years ago non or mildly pathogenic, while this haplotype has recently been considered to alter the protein function of BRCA1 [29]. In another patient, two variations were found: p.F486L and p.N550H. We considered the result as pathogenic. Analyzing the cosegregation of the variations with the cancer phenotype within the family can help resolving this problem, but the high cost and the reluctance of the families to undergo the screening prevent proceeding with this study.

To date, no other mutational analysis on breast cancer has been conducted in Lebanon. This report helps determining the spectrum of *BRCA1/2* point mutations in our country and must be the beginning of other studies with a larger cohort in order to determine the prevalence of the mutations in the general population and to check for the existence of founder mutations given that each of the following mutations p.C44F and p.W1815X were found twice in this cohort. It will be also interesting to evaluate the differences of *BRCA1/2* mutations frequencies among women of diverse ethnicities in Lebanon (Muslims and Christians) or more importantly, whether ethnicity should be taken into account in *BRCA1/2* risk assessment. The answers are important considering the eligibility for genetic testing, and the adoption of targeted prevention strategies.

## Competing interest

The authors declare that they have no competing interests.

## Author’s contributions

NJ, JN, EC, NS, NU, DSL, YJB, AM carried out the molecular genetic studies, participated in the sequence alignment ; DSL, YJB, AM drafted the manuscript. YJB and AM participated in the design of the study All authors read and approved the final manuscript.
